# Do changes in women's household status in Nepal improve access to food and nutrition?

**DOI:** 10.1111/mcn.13374

**Published:** 2022-05-25

**Authors:** Nadia Diamond‐Smith, Mahesh Puri, John Neuhaus, Sheri Weiser, Suneetha Kadiyala

**Affiliations:** ^1^ Department of Epidemiology and Biostatistics University of California San Francisco California USA; ^2^ Center for Research on Environment, Health and Population Activities Kathmandu Nepal; ^3^ Department of Medicine University of California San Francisco California USA; ^4^ London School of Hygiene and Tropical Medicine London UK

**Keywords:** dietary diversity, household eating patterns, labour force participation, pregnancy, South Asia, young, newly married women

## Abstract

Women's nutritional status remains poor in South Asia, impacting maternal and infant health outcomes. Women's household status is also low, as evidenced by eating behaviours. We started with triadic qualitative interviews with newly married women, husbands and mothers‐in‐law to explore the link between women's status and eating patterns, followed by longitudinal data from a cohort of 200 newly married women in rural Nepal to measure associations over time. Quantitative data were collected every 6 months for 18 months (four rounds of data) between 2018 and 2020. Interviews suggested that household relationships, women's status, and how much and what types of food she was given were intricately linked. Using mixed effects logistic regression models, we explore the association between markers of changing women's status (becoming pregnant, giving birth and working outside the home) on two outcomes (eating last always/usually and achieving minimum dietary diversity). We also explore for interaction between women's status and household food insecurity. Pregnancy increases women's dietary diversity, but this is not sustained post‐partum. Women who work outside the home are less likely to eat last in the household. Food insecurity is associated with both the order of household eating and dietary diversity. Interactions between food insecurity and giving birth suggested that women who give birth in food insecure households are more likely to eat last in the household. Changes in women's household status are associated with some improvements in dietary diversity and order of household eating, but the associations are not long‐lasting and depend on food security status.

## Key messages

- Newly married women in rural Nepal face low dietary diversity, often eat last, and this does not improve over the first few years of marriage.
- Although pregnancy does improve dietary diversity, this does not remain post‐partum.
- Working outside the home does appear to lead women to not eat last as frequently.
- Food insecurity is the most important factor contributing to dietary diversity and order of household eating.

## Graphical abstract

Women's status measured by pregnancy or work outside the home may be associated with access to food and nutrition. We find that changes in women's household status are associated with some improvements in dietary diversity and order of household eating, but the associations are not long‐lasting and depend on food security status.

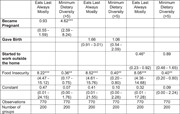

## 1 INTRODUCTION

Rates of women's undernutrition in South Asia, including in Nepal, remain staggeringly high, which could be due in part to poor access to food and poor diet quality. Women, especially pregnant women, in South Asia, including Nepal, have inadequate consumption of micronutrients (Henjum et al., 2015; Jiang et al., 2005). Inadequate micronutrient consumption, combined with poor weight gain in pregnancy, is associated with adverse maternal and infant outcomes (Strauss & Dietz, 1999). In Nepal, about half of households are food insecure, and 56% of women of reproductive age live in food insecure households (S. Pandey & Fusaro, 2020). Household food insecurity has been shown to be associated with women's dietary diversity and order of household eating (Kang et al., 2019; A. Singh et al., 2014; D. R. Singh et al., 2020).

Gender inequalities in access to food are drivers of these trends, with research in Nepal finding that women receive less food and lower quality food than men (Gittelsohn et al., 1997; Gittelsohn, 1991; H. A. Harris‐Fry, Paudel, Shrestha, et al., 2018). A systematic review of intrahousehold food allocation in South Asia described how the distribution (or inequitable distribution) of food between men and women within households is affected by household factors such as wealth, education, caste/religion and, importantly, food security, with distribution being more unequal in more food secure households (H. Harris‐Fry et al., 2017). Household gender norms are also important factors contributing to dietary diversity in Nepal (Morrison et al., 2021).

Most households in rural Nepal are coresident, with couples, especially when newly married and for the first few years of marriage, living with the husband's parents (in‐laws of the women) (Joshi, 2019). Thus, newly married women often find themselves living in a new household whose family members they do not yet know and where they are at the lowest status in the household hierarchy. Decision‐making is often at the household or couple level, including women's health care (such as family planning) and household issues such as eating (Morrison et al., 2018; Underwood et al., 2020). Relational factors within the household also contribute to a woman's status such as differences in individual's incomes, social status and household relationships (H. Harris‐Fry et al., 2017).

For women's optimal nutritional outcomes, women must have access to both diverse (quality) and adequate (quantity) nutritious foods. Dietary diversity has been found to be low among pregnant, lactating and young women in Nepal (H. A. Harris‐Fry, Paudel, Harrisson, et al., 2018; Henjum et al., 2015; D. R. Singh et al., 2020; J. K. Singh et al., 2019). A woman's status in her household has been found to be associated with dietary diversity. A recent study in Nepal found that as women transition from their parents' home into the husband's home at the time of marriage, the diversity of foods they eat decreases (N. Diamond‐Smith, Shieh, et al., 2020). Other markers of women's status within her household, including decision‐making power and employment, have been shown to be associated with dietary diversity in Nepal (Shrestha et al., 2021).

The other factor contributing to the overall nutritional status is how much food is consumed. As discussed above, household constraints such as food insecurity affect how much food is consumed, and women may be more affected by food insecurity compared with men (and younger women more than older women or children) (Kang et al., 2019; A. Singh et al., 2014; D. R. Singh et al., 2020). Another factor that has been found to be associated with the quantity of food consumed is the order of household eating. Past research in Nepal and other parts of South Asia has documented common practices of the daughters‐in‐law cooking and serving the rest of the family, and eating last in the household (Gittelsohn et al., 1997; Gittelsohn, 1991; H. A. Harris‐Fry, Paudel, Shrestha, et al., 2018; Morrison et al., 2018, 2021). This can leave women with not enough food overall and/or only certain, potentially less nutritionally diverse foods after everyone else has eaten. Order of household eating has been found to be associated with the quality and quantity of foods consumed in other South Asian countries (Barker et al., 2006; Palriwala, 1993). At the intersection of these factors—previous research in Nepal has also suggested that it is when households face food insecurity that the order of household eating makes an impact on the actual food consumed (Gittelsohn, 1991).

Despite these studies showing that women's household position affects their health‐care use or quantity and quality of their food consumption, we know little about specific events or activities that might change women's status in terms of eating order or diversity of foods consumed. Not all types of status are changeable of course. For example, a woman's education is unlikely to change after marriage in this setting (although that may be shifting with more access to higher level education over time). However, some components can change. Past research in Nepal found that women who were working outside of the home were more likely to use prenatal care (Furuta & Salway, 2006). Thus, working outside the home might lead to improvements in her status and subsequent access to food. There are also other pathways through which work outside the home might impact consumption, such as increased income, however, these are unlikely to impact the order of eating. On the other hand, it is possible that women who work might have less time to cook or eat, and might eat more street food or snack foods, lowering the quality or quantity of food they consume, although we were unable to find literature on this in Nepal.

As another example, a woman's ability to get pregnant, and give birth, especially to a son, is very important culturally in South Asia (Brunson, 2010; Rai et al., 2014). Subsequently, after women become pregnant or give birth they may be more able to access resources, such as high‐quality foods. On the one hand, we expect women's access to food and consumption of a more nutritious diet to increase during pregnancy because, given the importance of maternal nutrition in this period, it has been a focus in prenatal care, community outreach, government programmes and mass media. The Government of Nepal implements several programmes to address malnutrition. Even though the emphasis of these programmes is mainly on improving the nutritional status of infants and young children, almost all of these programmes have implications for addressing maternal undernutrition, either directly or indirectly. Recent qualitative research in the plains of Nepal found that when some women became pregnant they were prioritised and ate first, however, many women still cooked, served food and then late last, sometimes leading to not eating enough, even while pregnant (Morrison et al., 2021). On the other hand, pregnancy may lead to morning sickness or food aversions, leading women to eat less or not eat certain foods, leading to lower dietary diversity (Christian et al., 2006; Harding et al., 2017). Less is known about women's eating in the post‐partum period, a time when women actually have nutritionally higher requirements than in pregnancy. One could hypothesise that, however, given the emphasis on nutrition in pregnancy, nutrition post‐partum might actually appear to decline as it stabilises back to the prepregnancy 'normal' patterns. Thus, more research, including longitudinal quantitative studies, is needed about what happens to women's food consumption patterns during pregnancy, and after pregnancy as well.

The aim of this article is, first, to explore qualitatively the link between women's access to food and their household status, especially related to two factors that are known to contribute to a woman's status: (1) 'proving' fertility and (2) contributing an income. Next, we quantitatively analyse if changes in these two factors lead women to eat last in the household less frequently and/or have a more diverse diet.

1. We hypothesise that women who become pregnant and then give birth would be more likely to have a diverse diet and less likely to eat last. This is because women who are about to or have recently produced a child may be more 'valued' and also receive higher dietary diversity and not eat last.
2. We hypothesise that having a job that provides income will increase a woman's negotiating power, while also potentially contributing to the household's ability to spend money on food, so it should increase dietary diversity and lead her to not eat last.

Given household constraints on access to food due to food insecurity, it is possible that a change in status might only be able to improve things for a subset of women. There is mixed evidence as to the association between food security and nutritional outcomes in Nepal (Nisar et al., n.d.; A. Singh et al., 2014; D. R. Singh et al., 2020). Previous studies have found that food insecurity modifies the relationship between factors associated with women's household status and family planning use in Nepal, suggesting that it can act as a modifier (N. Diamond‐Smith et al., 2017). Thus, it is important to consider and explore how women's access to food/nutrition and their status differs by the food security level of the household.

In this article, we present findings from an exploratory mixed‐methods study, including formative qualitative research with 20 newly married households and an 18 months longitudinal survey with 200 newly married women. The exploratory qualitative study helped us to understand perspectives about the link between household eating patterns and women's status, and the quantitative study then explored how changes in women's status do in fact impact her access to diverse diets and the order of household eating.

## 2 METHODS AND MEASURES

### 2.1 Formative qualitative study

We collected in‐depth qualitative interviews with 20 household triads (newly married women, their husbands and mothers‐in‐law, for a total of 60 interviews) in 2017. Women were recruited from Nawalparasi district, which is on the border with India, in the Terai (plains) region of Nepal. It was selected because it has lower levels of women's empowerment than other parts of Nepal (Acharya et al., 2010). Eligibility included being married in the last 4 months, age 18–25 and living in the same household as their mother‐in‐law. Details of this study, the data collection and the analysis approach are described elsewhere in detail (N. Diamond‐Smith, Plaza, et al., 2020; N. G. Diamond‐Smith et al., 2019). In summary, the selection of villages was done in partnership with our district advisory committee and after a mapping of selected villages to identify newly married households, permission was sought from the household head, after which consent was sought from the newly married women, and after that, from her husband and mother‐in‐law. A total of 34 households were identified, 28 were eligible, and from there 20 of the first 21 randomly approached agreed to participate. The questionnaire was developed by both the local and international teams, tested among a comparable population of women, and edited accordingly. We interviewed participants until data saturation was reached, which was agreed upon through ongoing discussions and concurrent analysis among team members while data collection was ongoing. As the interviews were exploratory in nature, the team felt saturation was reached when common themes were emerging and relatively few new themes arose. Interviews were conducted by researchers of the same age and sex as respondents, who were from similar communities and spoke the local language, to try to address any potential discomfort for participants.

#### 2.1.1 Qualitative analysis

We used a thematic approach to our analysis, which was guided by a codebook developed first based on a priori codes, and then was added to through an iterative process (Fereday & Muir‐Cochrane, 2006). Specifically, as has been described elsewhere, coders coded a small set of interviews separately, then met to discuss discrepancies and suggest edits and additions to the codebook (N. Diamond‐Smith, Plaza, et al., 2020; N. G. Diamond‐Smith et al., 2019). Discrepancies were discussed until consensus was reached, and this process continued until it was agreed that the codebook was complete. Additional themes emerged, and related codes were added to reflect those themes. Coding was conducted by two researchers in the United States and one in Nepal. Given the triadic nature of the interviews (households), after each interview was coded individually, interviews were then reread as intact triads (newly married woman, husband and mother‐in‐law). Additional coding was also conducted at this point for themes that had not arisen in the individual‐level coding process. We used the qualitative software Atlas‐ti for this process. This article only presents findings from the interviews with newly married women.

### 2.2 Quantitative longitudinal survey

Between 2018 and 2019, four rounds of cohort data were collected over 18 months (every 6 months) from 200 newly married women living in two municipalities (one rural and one urban municipality that overlapped with the municipalities from the qualitative phase) of Nawalparasi district of Nepal. Eligibility at baseline included being married in the last 4 months before baseline, age 18–25, and living in the same household as their mother‐in‐law. We screened 18,906 households in two municipalities (one rural and one urban), identified 302 eligible participants and selected 200 participants randomly. A total of 202 women were approached—Two women were not allowed to participate in the study by their family members and were replaced by the nearest eligible women in the same area to reach a final sample of 200. This sample was estimated to measure a difference in child nutritional outcomes by comparing food secure and food insecure households (the primary aim of the parent study). Four trained female interviewers interviewed participants in their homes.

Questionnaires were first developed in English and then translated into Nepali. Questionnaires were pretested with 10 women in the outskirts of Kathmandu and necessary modifications were made before its actual use. Questionnaires were programmed into data collection software named the Kobo tool. Data were collected between February 2018 and October 2019. Research assistants obtained written informed consent and conducted survey interviews in person in a private space in participants' homes.

Both phases of this study were approved by the Nepal Health Research Council and the ethics committee of the University of California, San Francisco.

#### 2.2.1 Quantitative measures

Given the longitudinal nature of the data and analysis, variables were created for each of the four time points for all of the women's status‐related measures, eating behaviour measures and household food security measures. Baseline measures of the sociodemographic indicators were included.

##### 2.2.1.1 Women's status related measures

We explore the impact of three potential changes in women's status within the household, all specified as binary variables: becoming pregnant, giving birth and starting to work, all measured within the last 6 months (between survey time points). Specifically, we measure if women became pregnant, if a woman gave birth in the last 6 months, and if she started to work a job outside of the home in the last 6 months.

##### 2.2.1.2 Women's eating behaviours

We explore two main outcomes. The first is a woman's reports of how often she eats last in the household: never, rarely, sometimes, usually, all of the time. For our analysis, we constructed a binary variable combining women who report they ate last always or usually (1) with women who reported never, rarely and sometimes eating last (0). Our rationale was based on preliminary qualitative interviews with this population that suggested that always or usually eating last was indicative of 'low' household status and less access to food. Our second outcome was a measure of dietary diversity. Women were asked how frequently they ate a list of 10 food groups: cereals/grains/tubers, Vitamin A rich fruits/vegetables, green leafy vegetables, other fruits, other vegetables, meat/poultry, eggs, fish/seafood, milk and milk products and pulses. Based on previous work by our local research partners and with input from the community, we combined nuts with beans, peas and lentils in our questionnaire; thus the items differed slightly from the original Minimum Dietary Diversity for Women (MDD‐W). We created a binary variable with women who reported eating five or more of these in the last day coded as having a diverse diet (1) and those eating fewer than five of these as a not diverse diet (0). This is based on the recommendations for a binary measure of Minimum Dietary Diversity for Women, using the list method (FAO and FHI 360, 2016).

##### 2.2.1.3 Household food security

Food security was measured using the Household Food Insecurity Access Scale (HFIAS), which includes a set of nine items about experiences of food insecurity, uncertainty and anxiety, with answer choices of never, rarely, sometimes, often (Coates et al., 2007). Responses are then summed, and women are categorised into a score ranging from food secure, mildly food insecure, moderately food insecure and severely food insecure. From this, we made a binary variable indicating any versus no food insecurity, as has been done extensively in the literature, including in Nepal (Maxwell et al., 2014; S. Pandey & Fusaro, 2020; D. R. Singh et al., 2020).

##### 2.2.1.4 Sociodemographic characteristics

We include several individual and household level demographic variables which have been found in previous literature to be associated with our outcomes of interest, and which we found to differ by food security status. These include women's age (continuous variable ranging from 18 to 25), women's education (categorical < 6 years, 6–12 years, over 12 years), religion (Hindu vs. other), caste (Brahmin/Chhetri, Indigenous groups, Untouchables/minorities), and household wealth quintile. We also include an indicator for the type of marriage (arranged vs. love), because previous research has shown this to be associated with women's status in this setting (Niraula & Morgan, 1996). We acknowledge that marriage formation processes are more complex than this simple dichotomy, as we found in formative qualitative research in this same study population (N. G. Diamond‐Smith et al., 2019).

#### 2.2.2 Statistical analysis

First, we describe our sample, comparing the sociodemographics of women living in food secure compared with food insecure households. Next, we show the trends over time in our primary predictors: pregnancy, birth and working outside the home. After this we describe the trend over time in our interaction variable (food security) and our primary outcomes: eating last always or most of the time and dietary diversity. We also test the association between the two primary outcomes, using a simple univariable logistic regression model.

We assess the magnitude of the association of changes in a woman's pregnancy, birth and working status with changes in her eating behaviours using mixed effects logistic models (Diggle et al., 2013). This allows us to estimate the magnitude of the effect of a change in the predictors on the outcome for an individual woman. In other words, for example, we can answer the question: Does an individual woman's likelihood of eating a more diverse diet change when she gives birth? These models included repeated measures of eating patterns as the outcome, pregnancy, birth or working status as the primary predictors and a random effect for women. The regression coefficient of the primary predictor measured the magnitude of the change in a woman's probability of an eating pattern as her status changed. We fit models that included interactions between the primary predictors and food insecurity to assess whether the magnitudes of the effects of the primary predictors differed by food security status. We tested the statistical significance of the interactions using likelihood ratio tests. We present odds ratios for the effects of the primary predictors and associated 95% confidence intervals by food security status. We control for age, education, caste, religion, wealth quintile, type of marriage (love/arranged) and household food security. Analysis was conducted using STATA version 15 (StataCorp, 2017).

As food security is a subjective measure we also explore the association between our three status variables (pregnant, recent birth and work outside the home) and food insecurity, also using mixed effects logistic regression models controlling for the same sociodemographics described above. The goal of this was to check that women's status did not change due to food insecurity, for example, that women were not more or less likely to get pregnant or work outside the home in food secure/insecure households.

## 3 RESULTS

### 3.1 Formative qualitative findings

Food was the main way that respondents described being shown love and showing love in return. One newly married woman described “My mother‐in‐law and father‐in‐law love me like their own daughter… They care if I eat food or not. They tell me to eat food.”, while her mother‐in‐law said “After the marriage, I have a good relation with her [daughter‐in‐law]. She cooks food and serves me with all the love.”

However, women who lived in households with weaker or strained household relationships talked about not being asked if they ate and people not caring what they ate. Caring about eating was one of the main ways that women mentioned that their families could show that they care about their health, as one woman described:

*R: I don't feel like they care about my health. My mother‐in‐law spends her whole day outdoors so she never enquires about what I ate or how I am feeling. I meet my husband once a week, we mostly talk on phone when he is out of town and whenever he is in town we hardly talk about my health problems*….

*I: What do you think would be useful to do to help members of your household care about your health?*

*R: They should ask me what my current health condition is, inquire if I wish to eat something different. They should at least bring me those food that I wish to eat and take me to hospital if any emergency occurs*.

Order of household eating was also discussed, with respondents describing allowing a daughter‐in‐law to eat first as being a sign that she was cared for “They take care of my health. They bring food to my room. They ask me to tell them what I want to eat. Everyone loves me a lot. My father‐in‐law tells me that he will eat only after I have my meals.”

Many respondents described the importance of women being given more food while pregnant. If fact, as described by the respondent below, one of the main reasons seen for women to be given adequate food was so that they would produce a healthy baby.

*I: According to you, is it important for women to eat special food for their health?*

*R: Yes, it is important. They must eat milk, yoghurt, fruits, fish, meat, and eggs. These foods give energy to the body as a woman must do lots of work. They have to give birth to babies too. If the baby is born when her body is weak then the baby will also be born weak. A healthy mother will give birth to a healthy baby. For that, good food must be eaten from the start. There will be no tension for the family if her health is good*.

When asked about how her in‐laws would react to her having a child, another women described how her family would care for her and her baby:

*They will care more for me. They will suggest me to do things that are right for me. They won't allow me to carry heavy load and they will take care of my baby. They will buy food for me. Other family members will also help me in my work. They will take care of me and my baby*.

Financial concerns and instability were intricately linked to what women were provided to eat by their families, and what they felt able to ask for, especially given their status as newly married women:

*No one in the home has a good income. How can we eat in absence of good income? You need a good income and money to eat food of your wish. It's due to lack of money and income that I have not been able to eat food as desired…. You need to have money to buy as well. As I am newly married I feel shy to say about my likes and wants. If I even say who would bring these, you need to have money. So, it's difficult…. How can you eat without money? No one has told me to eat anything for my health till today*.

Aside from discussions about showing love through food and feeling cared for by being given food, another common marker of women's status (or lack thereof) was her inability to leave the house in the early days of marriage. Lack of mobility was frequently lamented by the women, and giving birth was seen as an event that could allow women more freedom of movement, as described below.

*In our community, you are not allowed to go outside home until you give birth to 1‐2 children…new daughter in laws are not allowed to go out of home, you need stay at home although you wish to go outside…. You don't get to do things as you want to*.

### 3.2 Quantitative longitudinal survey findings

Of the 200 participants who completed the baseline survey, 192 completed Round 2 of the survey, 191 completed Round 3 of the survey and 187 completed Round 4 of the survey. Most 183 (92%) participants completed all four rounds of the survey. Those lost to follow‐up were mostly lost due to migration, which is common in this area. Women were on average 20 years old, with women in food insecure households being younger (Table 1). Most (69.7%) of women had 6–12 years of school, with a higher proportion of women in food insecure households having less education. Most (86%) of women were Hindu, and this proportion was higher in food secure households. About half (53.6%) of the women in our sample were from Indigenous caste groups, 22.1% Brahmin/Chhetri and 24.3% from the so‐called minority/untouchable groups, with a greater proportion of food insecure households being from less advantaged caste groups (indigenous and the so‐called minority/untouchables. Finally, 70% of women had arranged marriages, and this was significantly higher in food insecure households.

**Table 1:** Background characteristics at baseline by household food security status at baseline.

	Food secure		Food insecure		Total	
	*N*	%	*N*	%	*N*	%
Food secure Households (binary)	93	100	107	100	200	100
Age at baseline mean, range***	21.33	18–25	19.67	18–24	20.44	18–25
Education***						
<6 years	1	1.1	32	29.9	33	16.5
6–12 years	68	73.1	69	64.5	137	68.5
Over 12 years	24	25.8	6	5.6	30	15
Religion**						
Hindu	82	88.2	90	84.1	172	86
Other	11	11.8	17	15.9	28	14
Caste***						
Brahmin/Chhetri	40	43	5	4.7	45	22.5
Indigenous group	41	44.1	65	60.7	106	53
Untouchables/minority group	12	12.9	37	34.6	49	24.5
Wealth quintile***						
Lowest	2	2.2	38	35.5	40	20
Second lowest	7	7.5	33	30.8	40	20
Middle	20	21.5	20	18.7	40	20
Second richest	32	34.4	12	11.2	44	22
Richest	32	34.4	4	3.7	36	18
Type of marriage***						
Love	35	37.6	24	22.4	59	29.5
Arranged	58	62.4	83	77.6	141	70.5

** *p* < 0.01.

*** *p* < 0.001.

Eight percent of women were pregnant within 4 months of marriage (baseline/Round 1), 37% at 6 months (Round 2), 25% at a year (Round 3) and 11% at 18 months (Round 4) (Table 2). Births followed this trend, with the peak number of women having recently given birth between Rounds 2 and 3. The proportion of women working outside the home declined over time, from 26% (*N* = 52) in Round 1 to 19.3% (*N* = 36) in Round 4.

**Table 2:** Change over time in status‐related predictors: Became pregnant, gave birth, started working outside the home.

	Round 1		Round 2		Round 3		Round 4	
	No.	%	No.	%	No.	%	No.	%
Became pregnant								
No	184	92	121	63	144	75.4	166	88.8
Yes	16	8	71	37	47	24.6	21	11.2
Recently gave birth								
No	199	99.5	183	95.3	118	61.8	150	80.2
Yes	1	0.5	9	4.7	73	38.2	37	19.8
Started working outside the home								
No	148	74	167	87	156	81.7	151	80.7
Yes	52	26	25	13	35	18.3	36	19.3
Total	200	100	192	100	191	100	187	100

**Figure 1:**
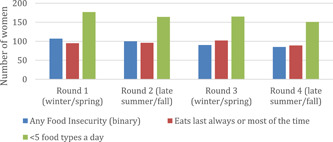
Trends over time in eating patterns and stressors: Eating last, dietary diversity and food insecurity.

Round 1 occurred in late winter/early spring (Feb/March), Round 2 in late summer/early fall (September/October), Round 3 in late winter/early spring and Round 4 in late summer/early fall (Figure 1). The proportion of women living in food insecure households increased over time (from about 14% in Round 1 to 39% in Round 4), as did the proportion living in food secure (about 47% in Round 1 to 55% in Round 4) (Table 3). Over time, the proportion of women who reported that they ate last all the time increased (25% in Round 1 up to 35.8% in Round 4), with the biggest jump between Round 1 to Round 2, and then a leveling off. Overall, a greater proportion of respondents ate fewer than five different food groups in a day (85.3%), and this generally decreased over time, with the biggest drop between Rounds 1 and 2. There was a strong, significant association between eating last and minimum dietary diversity, with eating last decreasing the odds of meeting minimum dietary diversity (OR = 0.18, 95% CI = 0.07–0.46, Round 4 data).

**Table 3:** Change over time in food insecurity, order of household eating and dietary diversity

	Round 1 (late Winter/Spring)		Round 2 (late summer/fall)		Round 3 (late Winter/Spring)		Round 4 (late summer/fall)		Total	
	No.	%	No.	%	No.	%	No.	%	No.	%
Food security status										
Food secure	93	46.5	92	47.9	101	52.9	102	54.5	388	50.4
Mildly food insecure	36	18	11	5.7	7	3.7	3	1.6	57	7.4
Moderately food insecure	44	22	40	20.8	9	4.7	9	4.8	102	13.2
Severely food insecure	27	13.5	49	25.5	74	38.7	73	39	223	29
Any food insecurity (binary)	107	53.5	100	52.1	90	47.1	85	45.5	382	49.6
How often respondent eats last										
Never	76	38	57	29.7	41	21.5	42	22.5	216	28.1
Rarely	17	8.5	21	10.9	37	19.4	42	22.5	117	15.2
Sometimes	12	6	18	9.4	11	5.8	14	7.5	55	7.1
Usually	45	22.5	26	13.5	35	18.3	22	11.8	128	16.6
All of the time	50	25	70	36.5	67	35.1	67	35.8	254	33
Eats last always or most of the time	95	47.5	96	50.0	102	53.4	89	47.6	382	49.6
Dietary diversity										
<5 food types a day	177	88.5	164	85.4	165	86.4	151	80.8	657	85.3
≥5 food types a day	23	11.5	28	14.5	26	13.6	36	19.3	113	14.7
Total	200	100	192	100	191	100	187	100	770	100

Women who became pregnant women were more likely to achieve minimum dietary diversity (OR = 4.62, 95% CI = 2.59−8.24) (Table 4). Women who began working outside the home were less likely to eat last in their households (OR = 0.46, 95% CI = 0.23−0.92). There was no significant association between becoming pregnant and eating last; working outside the home and dietary diversity, or having recently given birth and either outcome. Across the board, food insecurity was associated with increased odds of eating last and decreased odds of achieving minimum dietary diversity.

**Table 4:** Multivariable mixed‐effects models exploring the association between three measures of women's status (pregnancy, birth and work outside the home) and two outcomes (eating last in the household and having minimum dietary diversity) (odds ratio, 95% confidence interval).

	Eats last always mostly	Minimum dietary diversity (>5)	Eats last always mostly	Minimum dietary diversity (>5)	Eats last always mostly	Minimum dietary diversity (>5)
Became pregnant	0.93	4.62***				
	(0.55–1.59)	(2.59–8.24)				
Gave birth			1.66	1.06		
			(0.91– 3.01)	(0.54–2.09)		
Started to work outside the home					0.46*	0.89
					(0.23–0.92)	(0.48–1.65)
Food insecurity	8.22***	0.36**	8.52***	0.40**	8.05***	0.40**
	(4.47–15.12)	(0.17–0.75)	(4.61–15.76)	(0.20–0.80)	(4.36– 14.88)	(0.20–0.80)
Constant	0.47	0.07	0.41	0.10	0.32	0.09
	(0.01–24.15)	(0.00–1.76)	(0.01– 21.55)	(0.00–2.28)	(0.01– 17.28)	(0.00–2.24)
Observations	770	770	770	770	770	770
Number of groups	200	200	200	200	200	200

*Note*: Controlling for age, religion, wealth, caste, religion, type of marriage (arranged/love).

* *p* < 0.05

** *p* < 0.01

*** *p* < 0.001.

We hypothesised that food insecurity could interact with the status variables to impact outcomes. Table 5 shows the likelihood ratio tests comparing models with and without interactions. The Wald test values for these likelihood ratio tests suggest that models with interaction models for giving birth and food security were significant for eating last (interaction *p* < 0.001) and marginally significant for minimum dietary diversity (interaction *p* = 0.04).

**Table 5:** Table likelihood ratio test results to test for interaction (Prob > *χ* ^2^).

	Eats last always of most of the time	Ate at least five different foods in the last 24 h
Became pregnant	0.09	0.34
Gave Birth	0.00	0.09
Started working outside the home	0.65	0.11

Thus we ran interaction models for giving birth and found that women who gave birth in food insecure households were more likely to eat last than women not giving birth (OR = 6.84, 95% CI = 2.08–22.29) while women who gave birth in food secure households were less likely to eat last (OR = 0.59, 95% CI = 0.24–1.46) (Table 6). There were no significant effects for the interaction for dietary diversity.

**Table 6:** The effect of giving birth and food insecurity on eating order and dietary diversity, mixed effects logistic regression models with interaction term (odds ratio, 95% confidence interval).

	Eats last always of most of the time	Ate at least five different foods in the last 24 h
Comparison no recent birth, living in food secure household		
Gave birth in food secure household	0.59	1.45
	(0.24–1.46)	(0.68–3.08)
Gave birth in food insecure household	6.84***	0.20
	(2.08–22.29)	(0.24–1.01)
Living in a food insecure household, no recent birth	6.11***	0.50
	(3.22–11.62)	(0.02–1.77)
Observations	770	770
Number of groups	200	200

*Note*: Adjusting for age, education, religion, caste, household wealth and type of marriage.

*** *p* < 0.001.

Our tests for an association between the status variables and food security found nonsignificant association between changes in status and women's reports of food insecurity (Supporting Information: Appendix A).

## 4 DISCUSSION

This study adds to other recent studies showing that the relationship between women's empowerment and nutritional outcomes is complex and that other household factors may contribute more to outcomes such as women's dietary diversity (Quisumbing et al., 2021). Our primary finding is that considering food insecurity is essential for understanding how changes in women's status in the household affect their eating patterns. Overall, young, newly married women's consumption of diverse foods declined somewhat over the first 18 months of marriage, while they also are more likely to eat last always or mostly over time. During the first few months and years of marriage women are increasingly likely to get pregnant and give birth, and also leave paid work. These hypothesised changes in status (proving fertility and providing income) do lead to some changes in the order of eating and dietary diversity—most consistently for giving birth. However, household food security status makes the biggest difference—suggesting that changes in status may have differential impacts depending on how food constrained the household is.

We do find that dietary diversity (eating at least five different foods in a day) increases for women when they become pregnant—likely a testament to intensive programming and outreach from community health workers aiming to improve dietary diversity in pregnancy. In food secure households, this effect remains after birth (with increased dietary diversity), but the opposite happens in food insecure households. We interpret this as indicating that while households understand the importance of providing dietary diversity to women while pregnant and providing them access to more diverse diets, this does not translate into longer term changes in the distribution of resources in the household. Therefore, current programmes targeting pregnant and post‐partum women need to be revisited to highlight the importance of nutrition and access to enough food for women in the post‐partum period. This is key because nutritional and caloric needs are high among lactating women, even higher in fact than in pregnancy, and important for maternal and infant health (Ford et al., 2020). Starting to work outside the home, which could raise a woman's status through increasing the income she brings home, was not associated with improvements in dietary diversity—if anything we have some evidence (nonsignificant) that it led to less dietary diversity. This contrasts with our hypothesis and other recent research in Nepal on the effect of cash transfers on women's dietary diversity (H. Harris‐Fry et al., forthcoming). However, research in nearby Bangladesh found similar results as we did, with women's earnings not being associated with their individual dietary diversity (Sinharoy et al., 2019). More research is needed on the role of work outside the home, women's status, their income and access to food.

Women who give birth in food secure households are less likely to eat last, whereas giving birth in food insecure households puts women more at risk of eating last. This may be an indicator that women had higher status while pregnant, but this disappeared, and even got worse, when they transitioned out of pregnancy when resources are limited. The reality of, or perception of, the increased burden due to having 'another mouth to feed' in households with food insecurity might contribute to this finding. Again, this highlights the need for more focus on post‐partum health and nutrition. On the other hand, a woman starting to work is associated with a decreased likelihood of her eating last in the household. Thus, we do have some evidence that some factors that positively change status do affect eating patterns, potentially through a pathway of increased bargaining power, agency and decision‐making power. Recent studies in Nepal have found that women's bargaining power was associated with child nutritional status, and extend the literature on seeing how this affects women's own nutrition (Kulkarni et al., 2021). However, we find that living in food insecurity holds women back from this advantage. In other words, food security appears to be necessary for women to be able to move up in the social order in terms of food allocation.

We have mixed findings of how pregnancy and birth affect the order of eating. It may be that the first pregnancy/birth is not adequate to gain status or to be sufficiently empowered to have access to food/nutrition—possibly women will gain this when they become older and have more than one child, especially a son. Son preference still exists in Nepal and is important to families, and thus women's status may increase with the birth of a son (Brunson, 2010). Unfortunately, we were not powered to assess the impact of the sex of the baby. More broadly related to household eating order, our study extends the previous literature to show that these patterns can change over time, and women may shift household eating order as they progress through various life events or as the household undergoes different struggles. In other words, it changes and may not be linear. More research could explore more about these patterns, decision‐making around the order of household eating and women's perspectives/lived experiences of these shifts.

It is also possible that other unobserved factors influence the order of eating and access to diverse foods. Past literature has described the cultural practice of newly married women (even after the first pregnancy or birth) eating last as them adhering to the tradition of respecting elders and showing love to children (Morrison et al., 2018, 2021). It is important to unpack this further. Our team's formative work with newly married women, their husbands and mothers‐in‐law, suggests that rather than this practice being a choice, it is that is actually reflective of relationships and power. Food is used as a sign of love has also been described elsewhere in South Asia (Chowbey, 2017). Thus, eating last or not enough are clearly markers of low status, and the findings from this analysis suggest that changes in status do affect the order of eating and diversity of diet.

Across the board women in food insecure households are more likely to eat last always or most of the time. This suggests that, when there are not enough resources, these newly married women suffer. Our findings support previous studies that are almost 30 years old in Nepal which also found an intersection between food insecurity and order of household eating and force us to rethink the order of household eating as simply a way of showing respect—clearly it is also a marker of status and gender equality (Gittelsohn, 1991). A recent analysis of data from India found that women who eat last have worse mental health, suggesting that there could be additional health impacts of this practice (Hathi et al., 2021).

Another interesting pattern is in food insecurity over time. While some households transitioned out of food insecurity, there was a bigger move in the proportion of households moving from mild/moderate food insecurity into severe food security. This divergence suggests that perhaps households who are struggling slightly with food insecurity are at risk of transitioning into higher degrees of food insecurity. At a country level, overall food insecurity remained relatively stable in the two most recent Demographic and Health Surveys in Nepal (2011 and 2016), with evidence of a decrease in the proportion of severely food insecure (Ministry of Health and Population (MOHP) [Nepal], Ministry of Health and Population MOHP Nepal New ERA ICF International Inc, 2012; Ministry of Health M. & New ERA/Nepal ICF, 2017). However, that data are not longitudinal, so more research is needed into pathways of food insecurity in these communities over time. It is also important to consider seasonality when interpreting food security trends (Broaddus‐Shea et al., 2018; R. Pandey & Bardsley, 2019), however, our data do not find clear evidence of food security trends being associated with seasonality (Rounds 1 and 3 were collected in the late winter/early spring and Rounds 2 and 4 in the late summer/early fall).

### 4.1 Limitations

Detailed, longitudinal data on nutrition and behaviours allows us to draw conclusions about how changes in one part of women's lives are associated with changes in dietary patterns and behaviours. However, this study has several limitations. The sample is relatively small (200 women) and all women lived in one district of Nepal. Thus, these findings are not generalisable to all married women or to other parts of Nepal necessarily. Small sample sizes did lead to some wide confidence intervals in our multivariable models. Another limitation is that we were not powered to look at differences by sex of the baby, among those that gave birth. We know from previous literature that the sex of the infant, particularly, having a male infant, is highly valued in this setting, and therefore women's status may be more likely to change after having a boy. Other measures of women's status may also have been more relevant to explore, including more detailed information on women's work (amount of income generated for example) or information about individual and household gender norms. Other measures of nutrition and food could also shed more light, including the quantity of food consumed, especially related to if eating last in the household does in fact equate to eating less food.

## 5 CONCLUSIONS

Young, newly married women in Nepal have low household status, as evidenced by eating last and eating less diverse diets. Factors that might increase status, in this case, pregnancy, giving birth and working outside the home can lead to women eating more diverse diets or not eating last. Food insecurity is important to consider, and women may be more likely to access food when other status‐related factors change in food secure households. This highlights the need to address food insecurity, or target food insecure households when aiming to improve preconception or early pregnancy nutrition.

Most worrisomely, while there is a bump in women's access to dietary diversity while pregnant, this disappears and even worsens, after the baby is born. This suggests that programmes and policies may be effectively raising awareness of the importance of pregnancy nutrition, but not post‐partum nutrition. Messaging and counselling need to be refocused to ensure that post‐partum women are having their nutritional and caloric needs met as well. Furthermore, programmes and interventions that aim to have a longer term impact on women's empowerment and access to diverse and adequate foods are needed, given that life course factors, such as giving birth and labour force participation, do not have meaningful and long‐lasting impacts on women's access to food.

## AUTHOR CONTRIBUTIONS

Nadia Diamond‐Smith: Designed research, analysed data or performed statistical analysis, wrote paper. Mahesh Puri: Conducted research, helped design research, provided feedback on paper. John Neuhaus: Analysed data or performed statistical analysis, provided feedback on paper. Sheri Weiser: Helped design research, provided feedback on paper. Suneetha Kadiyala: Helped analyse data and provided feedback on paper.

## CONFLICTS OF INTEREST

The authors declare no conflicts of interest.

## Supporting information

Supporting information.

## Data Availability

Data available on request from the authors.
