# Supplementary material for: Do changes in women's household status in Nepal improve access to food and nutrition?
Source: Matern Child Nutr. 2022 May 25;18(3):e13374. doi: 10.1111/mcn.13374 (PMC9218303; doi:10.1111/mcn.13374)
Supplement: Supplementary file 1 — Supporting information. [file MCN-18-e13374-s001.docx]

Appendix A: Sensitivity analysis of association between food insecurity and three primary predictors of interest (odds ratios, 95% confidence intervals)

|  | **Food Insecurity** | **Food Insecurity** | **Food Insecurity** |
| --- | --- | --- | --- |
| **Currently Pregnant** | 1.23 |  |  |
|  | (0.59 - 2.55) |  |  |
| **Recent Birth** |  | 0.47 |  |
|  |  | (0.21 - 1.07) |  |
| **Work outside the home** |  |  | 0.47 |
|  |  |  | (0.16 - 1.37) |
| **Age at baseline (years)** | 0.57** | 0.56** | 0.58** |
|  | (0.39 - 0.85) | (0.37 - 0.84) | (0.39 - 0.87) |
| **Woman’s education (categorical)** | 0.59 | 0.58 | 0.64 |
|  | (0.14 - 2.47) | (0.13 - 2.51) | (0.15 - 2.76) |
| **Religion (compared to Hindu)** | 1.13 | 1.13 | 1.14 |
|  | (0.16 - 8.27) | (0.15 - 8.48) | (0.15 - 8.44) |
| **Caste (higher is more disadvantaged)** | 17.75*** | 19.68*** | 17.64*** |
|  | (4.25 - 74.18) | (4.46 - 86.73) | (4.13 - 75.24) |
| **Wealth quintile at baseline** | 0.13*** | 0.12*** | 0.13*** |
|  | (0.06 - 0.30) | (0.05 - 0.29) | (0.05 - 0.30) |
| **Arranged marriage (compared to love)** | 14.83** | 16.07** | 14.05** |
|  | (2.74 - 80.20) | (2.82 - 91.68) | (2.55 - 77.51) |
| **Constant** | 24,721.85* | 44,994.16* | 21,340.04* |
|  | (5.09 - 120127221.69) | (7.28 - 277900111.15) | (3.97 - 114840654.51) |
| **Observations** | 770 | 770 | 770 |
| **Number of groups** | 200 | 200 | 200 |

*** p<0.001, ** p<0.01, * p<0.05
